# Diagnostic accuracy of interferon-gamma-induced protein 10 for differentiating active tuberculosis from latent tuberculosis: A meta-analysis

**DOI:** 10.1038/s41598-019-47923-w

**Published:** 2019-08-06

**Authors:** Xia Qiu, Ying Tang, Rong Zou, Yan Zeng, Yan Yue, Wenxing Li, Yi Qu, Dezhi Mu

**Affiliations:** 1 Department of Pediatrics, West China Second University Hospital, Sichuan University, Chengdu, China; 2 Key Laboratory of Obstetric & Gynecologic and Pediatric Diseases and Birth Defects of Ministry of Education, Sichuan University, Chengdu, China; 3 Ultrasonic Department, West China Second University Hospital, Sichuan University, Chengdu, China

**Keywords:** Diagnostic markers, Tuberculosis

## Abstract

Tuberculin skin test and interferon-gamma release assay are not good at differentiating active tuberculosis from latent tuberculosis. Interferon-gamma-induced protein 10 (IP-10) has been widely used to detect tuberculosis infection. However, its values of discriminating active and latent tuberculosis is unknown. To estimate the diagnostic potential of IP-10 for differentiating active tuberculosis from latent tuberculosis, we searched PubMed, Web of Science, Embase, the Cochrane Library, CNKI, Wanfang, VIP and CBM databases. Eleven studies, accounting for 706 participants (853 samples), were included. We used a bivariate diagnostic random-effects model to conduct the primary data. The overall pooled sensitivity, specificity, negative likelihood rate, positive likelihood rate, diagnostic odds ratio and area under the summary receiver operating characteristic curve were 0.72 (95% CI: 0.68–0.76), 0.83 (95% CI: 0.79–0.87), 0.32 (95% CI: 0.22–0.46), 4.63 (95% CI: 2.79–7.69), 17.86 (95% CI: 2.89–38.49) and 0.8638, respectively. This study shows that IP-10 is a potential biomarker for differentiating active tuberculosis from latent tuberculosis.

## Introduction

Tuberculosis (TB), one of the most serious infectious diseases, has exceeded acquired immune deficiency syndrome as a leading cause of death worldwide^1,2^. The World Health Organization reported approximately 1.04 million new TB patients and 1.674 million individuals died of TB in 2016 (Global TB report 2017)^2^. Furthermore, approximately 2000 million population in the worldwide are infected by *Mycobacterium tuberculosis (Mtb)* and have presumptive latent tuberculosis infection (LTBI)^3^. Although LTBI involves the absence of clinical TB symptoms, among all of the latent individuals, 10% of them have a risk of developing active TB^4,5^. Now, in order to eliminate TB, a major goal is to differentiate ATB from LTBI and treat LTBI^6^.

Routine diagnostic methods for ATB and LTBI include the evaluation of symptoms, chest X-rays and *Mtb* cultures^7^. Cough and low-grade fever, two of the TB symptoms, are non-specific in discriminating ATB and LTBI. Chest X-rays are also non-specific and should be used with other methods. Although specimen culture provides the most accurate diagnosis for ATB and LTBI, it is time-consuming and depends on specimen quality. Currently, the interferon-gamma release assay (IGRA) and tuberculin skin test (TST) are probably accurate immunodiagnostic methods for ATB and LTBI^8,9^. In particular, IGRA can overcome the limitation of TST, which lacks specificity among Bacilli Calmette Guerin (BCG)-vaccinated individuals^10^. However, both TST and IGRA have failed to correctly distinguish which stage of TB infection and cannot discriminate between ATB and LTBI^11–13^. The correct distinction between ATB and LTBI is critical for clinical treatment of ATB and LTBI. Considering these limitations, an additional immunodiagnostic test which can discriminate between ATB and LTBI are required.

Interferon-gamma-induced protein 10 (IP-10) is a cytokine which could persistently increase after TB infection. The expression level of IP-10 could increase one hundred times higher than IFN-gamma after TB infection, and it is not influenced by various ages, sexes, TB sites and presentations^14–16^. Several years ago, IP-10 has been investigated for its validity in differentiating ATB from LTBI^4,5,8^.

We urgently need to find a new biomarker to distinguish between ATB and LTBI. Therefore, we conducted a meta-analysis to evaluate the diagnostic potential of IP-10 for discriminating ATB from LTBI. More specifically, we did a meta-analysis to (1) establish the overall potential of IP-10 test for discriminating between ATB and LTBI; (2) assess the influence of various characteristics on diagnostic accuracy; and (3) identify potential factors associated with inconsistency in the studies.

## Results

### Literature research

**Figure 1:**
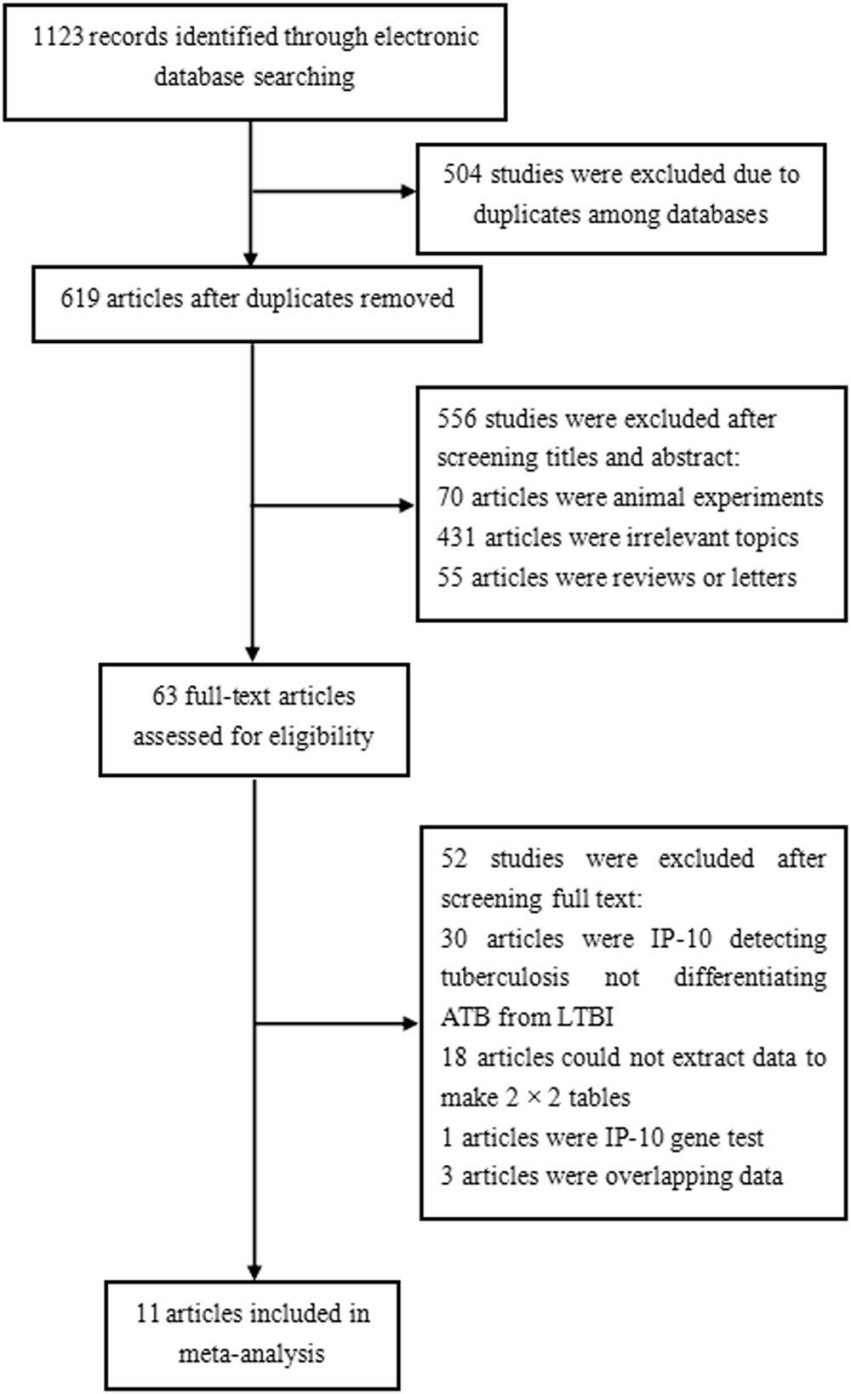
Flow chart of the identified and included articles. 1123 literature citations were identified from 8 databases (English databases: 925, Chinese databases: 198). After removing 504 duplicates, we read titles and abstracts and excluded 556 records (70 records focused on animal experiments, 431 records were irrelevant topics, and 55 records were reviews, abstracts or letters which beside the point). Ultimately, 11 articles including 15 trials were included.

1123 literature records were identified from 8 databases (English databases: 925, Chinese databases: 198) (Fig. 1). After removing 504 duplicates, we read titles and abstracts and excluded 556 records (70 records focused on animal experiments, 431 records were irrelevant topics, and 55 records were reviews, abstracts or letters which beside the point). Ultimately, 11 articles^4,5,8,17–24^ including 15 trials were included in this meta-analysis.

### Characteristics of the included studies

As shown in Table 1, characteristics of the 15 included trials are listed^4,5,8,17–24^. There were 702 participants and 853 samples involved. Thirteen included trials were described in English, and only two were described in Chinese^23,24^. The year of publication spanned 5 years, from 2012 to 2017. Ten (67%) trials were from upper-middle-income countries (UMICs), and five (33%) trials were from high-income countries (HICs). Reference standards were culture, clinical, radiological, tuberculin skin and interferon-gamma tests. The interferon-gamma test in our study included the QFT-GIT test, T-SPOT.TB test and IFN-γ ELISPOT test in Table 1. The numbers of ATB and LTBI patients, the ratio of males to females and the index test can also be seen in Table 1. The study design, HIV-infected condition, cut-off, sensitivity, specificity, TP, FP, FN, and TN of IP-10 were listed (Table 2).

**Table 1:** Main characteristics of studies included in the meta-analysis.

Author	Year	Country	World bank income classification	TB incidence rate per population	Participants (N)		index test (IP-10) condition	Reference standard
					ATB	LTBI		
Nonghanphithak D	2017	Thailand	UMIC	172 per 100,000	48	38	Unstimulated	culture, clinical, radiological, TST and QFT-GIT test
Nonghanphithak D	2017	Thailand	UMIC	172 per 100,000	48	38	TB Ag	culture, clinical, radiological, TST and QFT-GIT test
Yao XY	2017	China	UMIC	64 per 100,000	20	10	Unstimulated	culture, clinical, radiological, T-SPOT.TB and QFT-GIT test
Yao XY	2017	China	UMIC	64 per 100,000	20	15	Unstimulated	culture, clinical, radiological, T-SPOT.TB and QFT-GIT test
Wu J	2016	China	UMIC	64 per 100,000	25	36	Unstimulated	culture, clinical, radiological, TST and T-SPOT.TB test
Wu J	2016	China	UMIC	64 per 100,000	25	36	TB Ag	culture, clinical, radiological, TST and T-SPOT.TB test
Li XF	2016	China	UMIC	64 per 100,000	72	57	TB Ag	culture, clinical, radiological and T-SPOT.TB test
Jeong YH	2015	Republic of Korea	HIC	77 per 100,000	33	20	TB Ag	culture, clinical, radiological, TST and QFT-GIT test
Wergeland I	2015	Norway	HIC	6.1 per 100,000	6	23	Unstimulated	culture, clinical, radiological, TST and QFT-GIT test
Wergeland I	2015	Norway	HIC	6.1 per 100,000	59	11	Unstimulated	culture, clinical, radiological, TST and QFT-GIT test
Tebruegge M	2015	Australia	HIC	6.1 per 100,000	6	16	TB Ag	culture, clinical, radiological, TST and QFT-GIT test
Won EJ	2015	Republic of Korea	HIC	77 per 100,000	36	15	TB Ag	culture, clinical, radiological, TST and QFT-GIT test
Yang QT	2014	China	UMIC	64 per 100,000	20	17	TB Ag	culture, clinical, radiological and IFN-γ ELISPOT test
Chegou NN	2013	South Africa	UMIC	781 per 100,000	15	26	Unstimulated	culture, clinical, radiological, TST and QFT-GIT test
Wang S	2012	China	UMIC	64 per 100,000	28	34	Unstimulated	culture, clinical, radiological, TST and QFT-GIT test

UMIC: upper-middle-income countries, HIC: high-income countries, TB Ag: tuberculosis antigen, TST: tuberculin skin test, QFT-GIT: QuantiFERON-TB Gold In-tube, ELISPOT: enzyme linked immunospot.

**Table 2:** Baseline data of included studies.

Author	Year	IP-10 condition	Study design	HIV-infected	Cut-off (pg/ml)	Sensitivity (%)	Specificity (%)	TP	FP	FN	TN
Nonghanphithak D	2017	Unstimulated	case control	No	2812.5	87.5	78.9	42	8	6	30
Nonghanphithak D	2017	TB Ag	case control	No	27699	41.7	71.1	20	11	28	27
Yao XY	2017	Unstimulated	Identification cohort	No	1580	80	80	16	2	4	8
Yao XY	2017	Unstimulated	Replication cohort	No	3182	50	93.33	10	1	10	14
Wu J	2016	Unstimulated	cohort	Not reported	785.4	88	52.8	22	17	3	19
Wu J	2016	TB Ag	cohort	Not reported	1139	76	66.7	19	12	6	24
Li XF	2016	TB Ag	case control	No	8765.67	84.72	96.49	61	2	11	55
Jeong YH	2015	TB Ag	case control	Not reported	23780.88	69.7	100	23	0	10	20
Wergeland I	2015	Unstimulated	case control	Yes	2547	100	100	6	0	0	23
Wergeland I	2015	Unstimulated	case control	No	689	71	82	42	2	17	9
Won EJ	2016	TB Ag	cohort	No	145	63.9	80	23	3	13	12
Tebruegge M	2015	TB Ag	cohort	No	100	100	100	6	0	0	16
Yang QT	2014	TB Ag	cohort	No	1008	88	92	18	1	2	16
Chegou NN	2013	Unstimulated	cohort	Some	6768	73.3	80.8	11	5	4	21
Wang S	2012	Unstimulated	cross section	No	956.1	47.1	92.9	13	2	15	32

TP: true positive, FP: false positive, FN: false negative, TN: true negative.

### Quality assessment

The methodological quality of eligible articles was determined by QUADAS-2. In patient selection, bias was unclear for 6 studies, high in 1 study and low for 4 studies. Concerning index tests, only seven studies showed a low bias, and the remaining studies had unclear bias. Eight studies were deemed to have low bias in their reference standards, and three study showed unclear bias. Flow and timing bias was low in nine studies, unclear in one study and high in one study. Concerns related to patient selection were low for six studies and unclear for five studies. The applicability concerns were low for the index tests in nine studies and unclear in two studies. Regarding the reference standard, there was high concern for one study and unclear concern for ten studies. Major risks for bias pertained to participant selection, index test and reference standard whether in blind conditions.

### The overall diagnostic accuracy of IP-10

**Figure 2:**
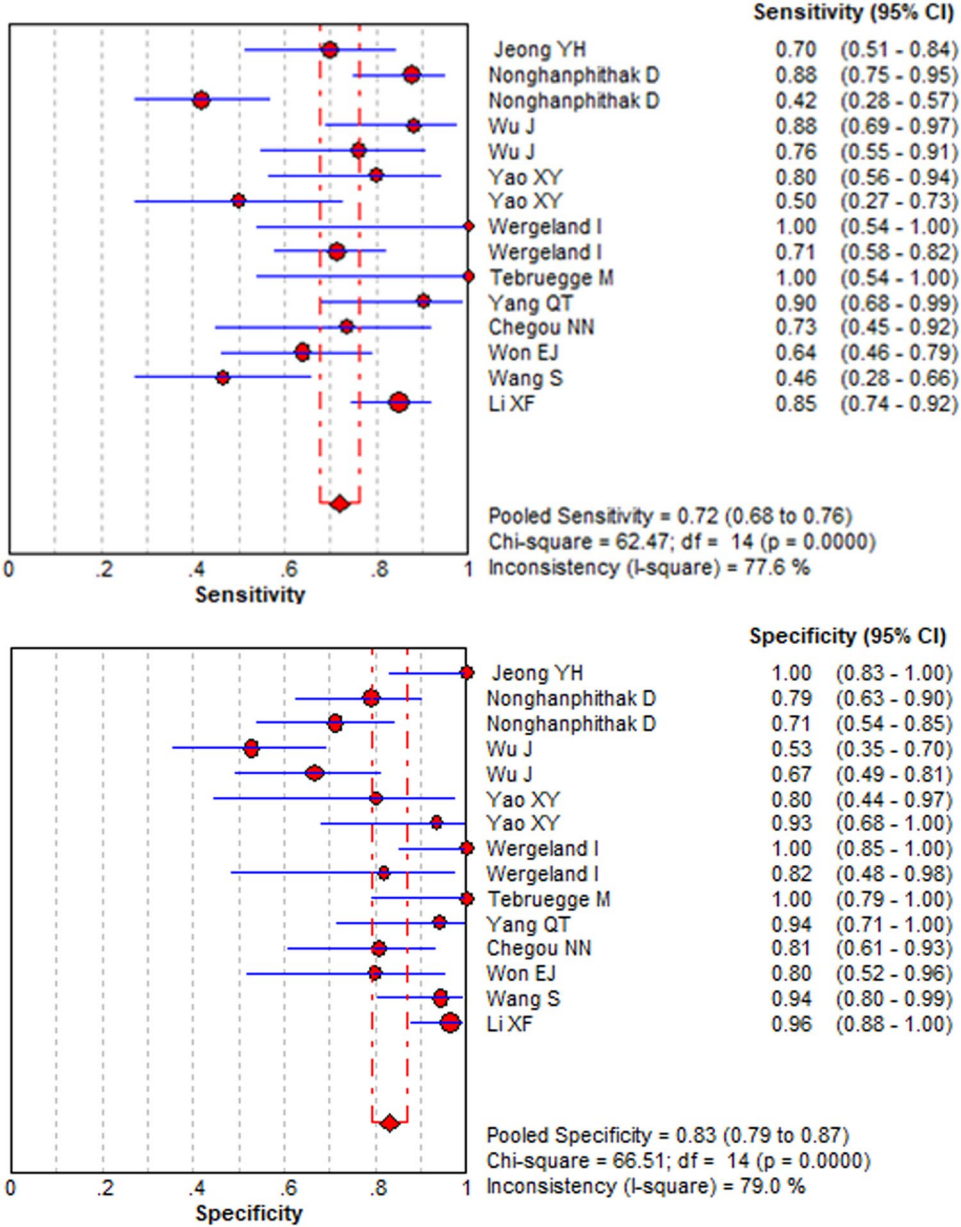
The forest plots of the pooled sensitivity and specificity of IP-10 for differentiating ATB from LTBI. The sensitivity ranged from 0.46 to 1.00 (pooled sensitivity: 0.72, 95% CI: 0.68–0.76, I^2^ = 77.6%); whereas, the specificity ranged from 0.53 to 1.00 (pooled specificity: 0.83, 95% CI: 0.79–0.87, I^2^ = 79.0%).

**Figure 3:**
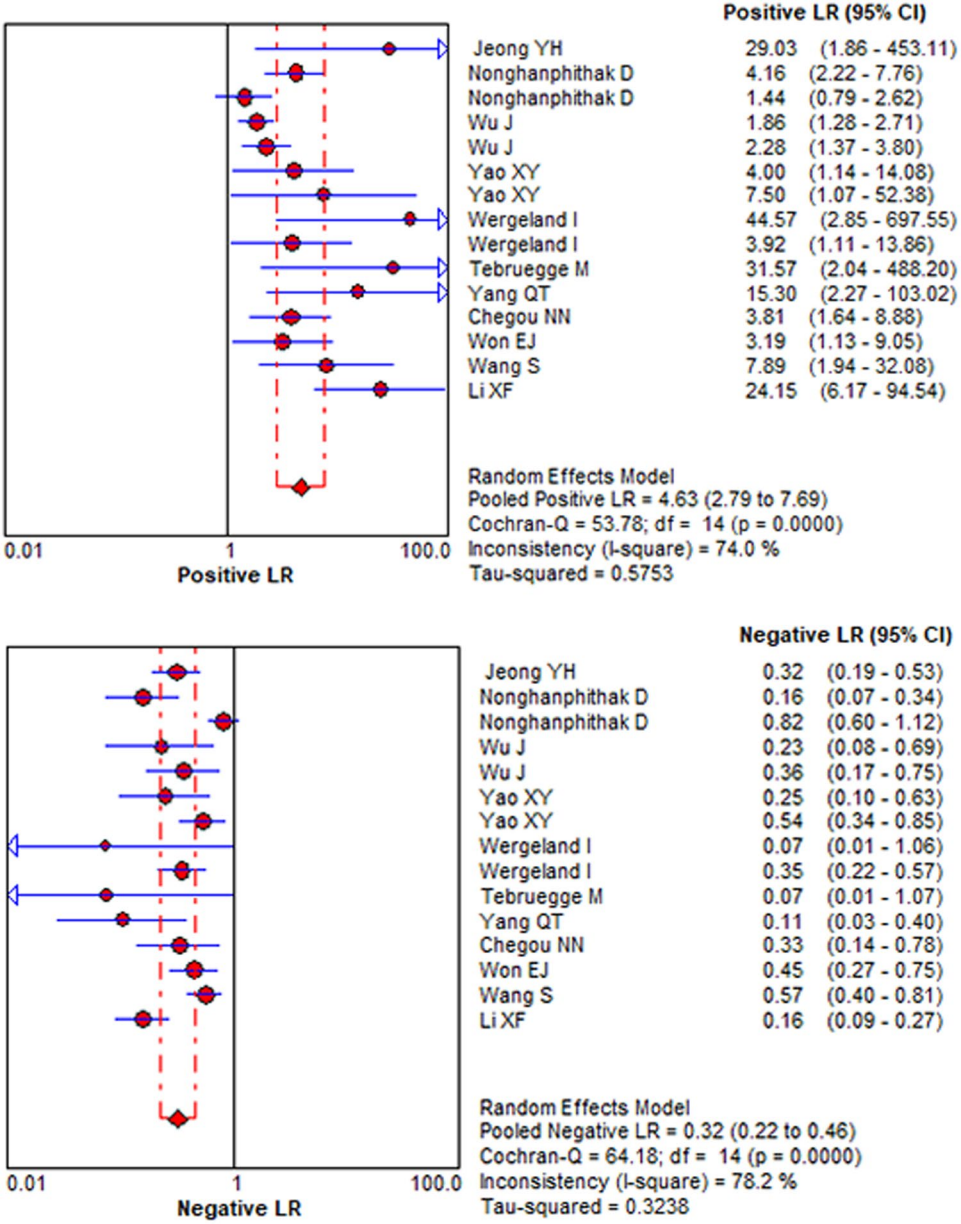
The forest plots of positive LR and negative LR of IP-10 for differentiating ATB from LTBI. The pooled PLR and NLR of IP-10 were 4.63 (95% CI: 2.79–7.69, I^2^ = 74.0%) and 0.32 (95% CI: 0.22–0.46, I^2^ = 78.2%), respectively.

**Figure 4:**
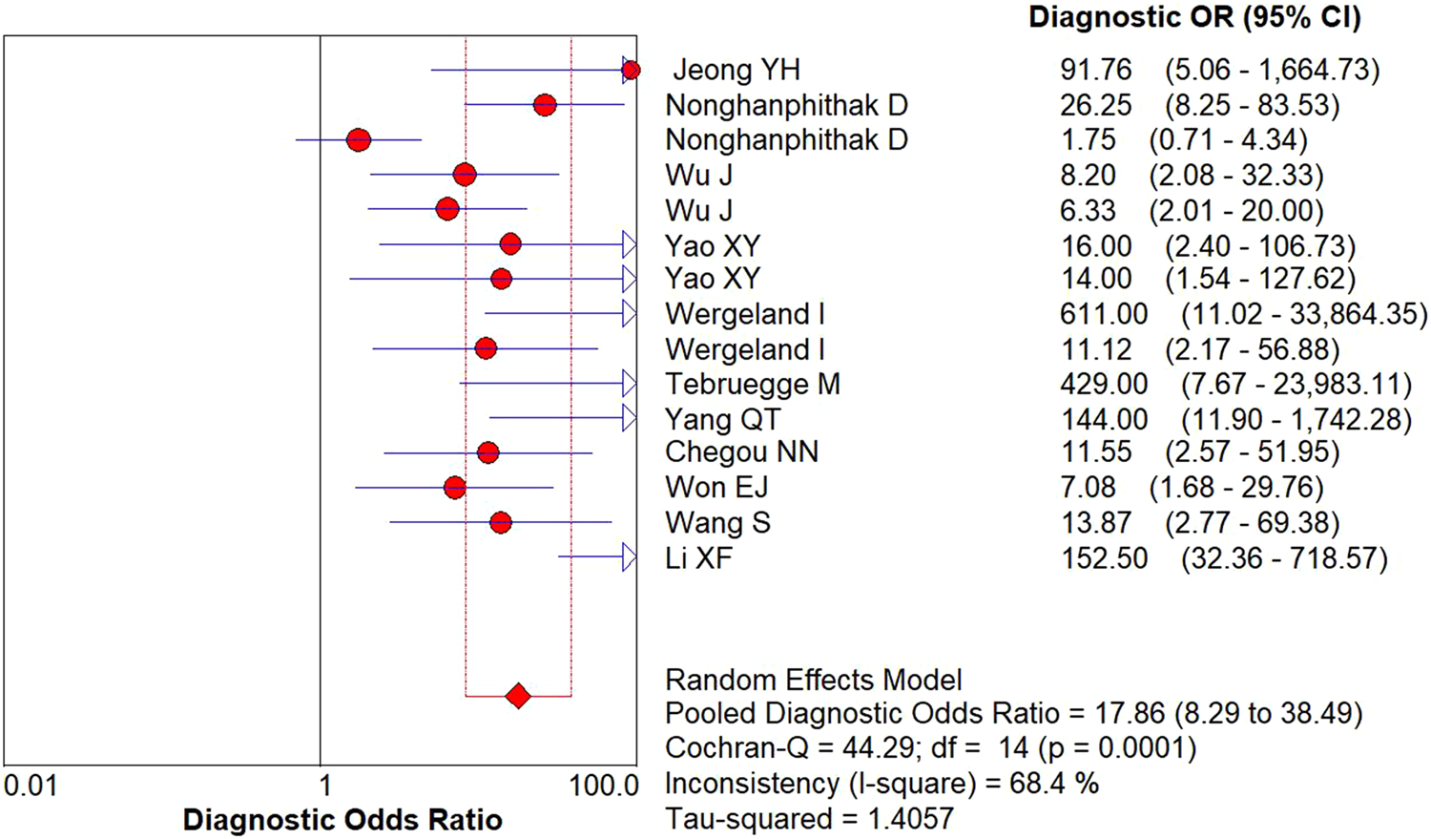
Forest plots of diagnostic odds ratio (DOR) of IP-10 for differentiating ATB from LTBI. The pooled DOR was 17.86 (95% CI: 2.89–38.49, I^2^ = 68.4%).

**Figure 5:**
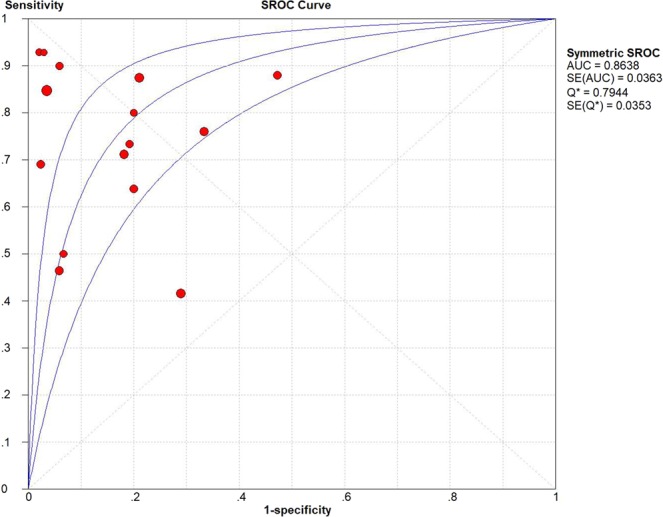
The curve for assessment of IP-10 for differentiating ATB from LTBI. The AUC and Q* value were 0.8638 and 0.7944, respectively. Summary receiver operating characteristic: SROC.

No threshold effect was found in this meta-analysis (Spearman correlation coefficient = − 0.229, P-value = 0.411). A random effects model was operated to detect IP-10 for differentiating ATB from LTBI. A total of 853 samples were detected. The sensitivity ranged from 0.46 to 1.00 (pooled sensitivity: 0.72, 95% CI: 0.68–0.76, I^2^ = 77.6%); whereas, the specificity ranged from 0.53 to 1.00 (pooled specificity: 0.83, 95% CI: 0.79–0.87, I^2^ = 79.0%) (Fig. 2). The pooled PLR and NLR of IP-10 were 4.63 (95% CI: 2.79–7.69, I^2^ = 74.0%) and 0.32 (95% CI: 0.22–0.46, I^2^ = 78.2%), respectively (Fig. 3). DOR, as a single indicator, could evaluate the discriminatory accuracy of the index test. In Fig. 4, DOR was 17.86 (95% CI: 2.89–38.49, I^2^ = 68.4%), presenting the ability of IP-10 for discriminating ATB from LTBI was relatively good. In addition, the AUC and Q* value were 0.8638 and 0.7944, respectively, which represented perfect discriminatory accuracy of IP-10 (Fig. 5).

### The potential heterogeneity

World Bank income classification, study design, HIV-infected condition, cut-off and IP-10 condition included in the meta-regression analysis were not potential sources of heterogeneity (P > 0.05). The diagnostic accuracy of IP-10 tests in high-income countries was 0.43 times higher than P-10 tests in upper-middle-income countries (RDOR = 0.43, 95% CI: 0.03–6.59; P = 0.4922).

### Subgroup analysis

Regarding the World Bank income classification, a total of 225 samples from high-income countries and 628 samples from upper-middle-income countries were detected. The sensitivity was similar in these countries (71% vs 72%). The specificity was higher in high-income countries comparing with upper-middle-income countries (94% vs 80%). The PLR of IP-10 in high-income countries was high (7.99 vs 3.91). The NLR was similar (0.35 and 0.32). The DOR and AUC are listed (Table 3).

**Table 3:** Subgroup analysis of the included study.

Subgroup		Studies	Sensitivity (95%)	Specificity (95%)	PLR (95%)	NLR (95%)	DOR	AUC
World bank income classification	HIC	5	0.71 (0.63,0.79)	0.94 (0.87,0.98)	7.99 (2.68,23.86)	0.35 (0.23,0.51)	33.69 (6.78,167.49)	0.8277
	UMIC	10	0.72 (0.67,0.77)	0.80 (0.75,0.84)	3.91 (2.27,6.74)	0.32 (0.20,0.51)	14.79 (5.98,36.60)	0.8576
IP-10	TB Ag	7	0.71 (0.65,0.77)	0.85 (0.80,0.90)	6.11 (2.20,17.02)	0.30 (0.16,0.58)	24.66 (5.15,118.18)	0.8456
	Unstimulated	8	0.73 (0.67,0.79)	0.81 (0.75,0.86)	4.08 (2.31,7.19)	0.34 (0.22,0.51)	15.36 (8.64,27.30)	0.8651
Study design	Cohort	8	0.75 (0.68,0.81)	0.76 (0.69,0.82)	3.40 (2.02,5.73)	0.34 (0.23,0.49)	12.09 (6.09,24.01)	0.8377
	Case control	6	0.73 (0.67,0.78)	0.88 (0.82,0.92)	6.69 (2.22,20.17)	0.28 (0.14,0.58)	29.15 (5.07,167.71)	0.8773
	Cross section	1	—	—	—	—	—	—
HIV-infected	Yes or some	2	0.81 (0.58,0.95)	0.90 (0.78,0.97)	9.52 (0.78,116.35)	0.24 (0.06,0.97)	53.54 (1.19,2403.79)	—
	No	10	0.70 (0.65,0.75)	0.87 (0.82,0.91)	5.33 (2.79,10.19)	0.33 (0.21,0.52)	19.31 (6.76,55.15)	0.8916
	Not reported	3	0.77 (0.67,0.86)	0.68 (0.58,0.78)	2.44 (1.15,5.18)	0.31 (0.21,0.47)	9.97 (3.20,31.08)	0.85
Cut-off	<2000 pg/ml	8	0.73 (0.66,0.78)	0.78 (0.71,0.84)	3.63 (2.07,6.37)	0.35 (0.24,0.50)	12.10 (6.19,23.65)	0.8343
	≥2000 pg/ml	7	0.71 (0.65,0.77)	0.88 (0.82,0.92)	6.38 (2.50,16.28)	0.31 (0.16,0.59)	25.64 (5.63,116.77)	0.8811

PLR: positive likelihood ratio, NLR: negative likelihood ratio, DOR: diagnostic odds ratio, AUC: area under the curve.

With respect to the condition of IP-10, 439 samples were used to measure TB Ag–stimulated IP-10, and 414 samples were used to measure unstimulated IP-10. The overall diagnostic performances of Ag–stimulated and unstimulated IP-10 were similar (Table 3).

Comparing the different study designs, a total of 338 samples were cohort studies, and 453 samples were case-control studies. There was only a cross-sectional study with 62 samples. The sensitivity was similar (75% and 73%). The specificity was higher in case-control studies than in cohort studies (88% vs 76%).

With respect to the HIV-infected condition, the diagnostic accuracy of IP-10 in HIV-infected patients was higher comparing with these HIV-noninfected and not-reported individuals. The sensitivity and specificity were higher in HIV-infected patients than HIV-noninfected and not-reported individuals (81% vs 70% and 77%, 90% vs 87% and 68%).

When the cut-off of IP-10 ≥ 2000 pg/ml, the specificity increased by 10% (88% vs 78%). The sensitivity was similar (71% and 73%).

### Publication bias

The results showed that the P-value obtained from the Deek’s funnel plot was 0.69, which indicated no striking publication bias.

## Discussion

TB is still a major public health issue worldwide, especially in young children and immunocompromised individuals^25,26^. Although 90% of LTBI individuals remain asymptomatic and do not progress to ATB, the timely and accurate detection and prophylactic treatment of LTBI individuals are important for controlling ATB worldwide^27^. As we all know, differential diagnosis of ATB and LTBI correctly is primary, current methods are strengthless. The search for new markers for discriminating ATB from LTBI is ongoing. Several studies showed that IP-10 might be a potential biomarker to discriminate ATB from LTBI^4,5,8,17–24^. Furthermore, IP-10 could monitor anti-TB treatment responses and improve TB diagnosis with HIV^28^. A new form (agonist/antagonist) of IP-10 could be detected in TB patients, and it may help IP-10 in TB diagnosis^29^.

In this study, we firstly conducted a meta-analysis to evaluate the overall performance of IP-10 as a new marker for discriminating ATB from LTBI. We found that IP-10 could be a potential marker for differentiating ATB and LTBI with moderate diagnostic value (sensitivity: 72%, specificity: 83%, AUC = 0.8638). The PLR of 4.63 and NLR of 0.32 suggested that IP-10 had good detection potential in discriminating between ATB and LTBI. No striking publication bias strengthened the correctness of the results.

We have previously reported the accuracy of IP-10 for diagnosing LTBI (Qiu, X. *et al*.)^30^. Compared with the report by Qiu, X. *et al*. 2018, this study had several main differences. First, the participants (patients and controls) were different. In the study by Qiu, X. *et al*. 2018, we compared LTBI individuals with non-TB populations. In this study, we compared ATB patients with LTBI individuals. Second, the conditions of IP-10 (index test) were different. In the study by Qiu, X. *et al*. 2018, we included only the Ag-stimulated IP-10. In this study, we included both Ag-stimulated and unstimulated IP-10, and the subgroup and meta-regression analysis for both Ag-stimulated and unstimulated IP-10 were performed. Finally, we searched more comprehensively than that in the study by Qiu, X. *et al*. 2018.

Currently, TST and IGRA are the most conventional tests for LTBI and ATB, which are as important as the assessment of symptoms, radiological and microbiological examination^8,9^. TST has been used for a long time, but it can show cross-reactivity among BCG-vaccinated individuals and lead to wrong judgement with the size of induration of the skin reaction^27^. Recent years, IGRA has been developed and can overcome some limitations of TST test. Currently, three IGRAs are used: QFT-GIT, T-SPOT.TB and QuantiFERON-TB Plus^10,31^. Although IGRA can be an alternative method of TST to detect ATB and LTBI, many original researches report poor IGRA accuracy in differentiating ATB from LTBI^17^. Nonghanphithak, D. *et al*. found that the IGRAs (QFT-GIT) discriminating between ATB and LTBI showed relatively low sensitivity (16.7%) for diagnosis of LTBI, while the sensitivity of IP-10 was 87.5%^5^. Wu, J. *et al*. reported that the sensitivity of IP-10 in discriminating ATB from LTBI was higher than IGRAs (T-SPOT.TB) (76% vs 52%) [4]. These results indicated that IP-10 is a helpful marker in discriminating ATB from LTBI. Even though Petrone, L. *et al*. reported the sensitivity (58%) and specificity (61%) were low in differentiating ATB and LTBI, they suggested that IP-10 was an alternative biomarker of QuantiFERON-TB Plus^32^.

Different World Bank income classification may lead to different performance of IP-10. Generally, the ATB and LTBI incidence rates were relatively low in developed countries. Although in subgroup analysis, when compared with upper-middle-income countries, the specificity was higher with high-income countries (94% vs 80%). The difference maybe the resource settings of IP-10 in high-income countries were much better, including high quality of detective equipment (commercial multiplex analyze human cytokines sets). World Bank income classification didn’t lead to heterogeneity (P = 0.4922). In further studies, high-TB countries and low-TB countries should be distinguished.

Regarding the condition of IP-10, we found that TB Ag-stimulated IP-10 had a similar diagnostic value as unstimulated IP-10. Previous studies showed that the level of IP-10 could increase one hundred times much more than IFN-gamma after TB infection, and not influenced by TB site and presentation^14–16^. In this study, we found that the heterogeneity was not influenced by IP-10 condition whether Ag-stimulated or not (P = 0.8032). In the next step, in order to find the best condition of IP-10, we also suggest that Ag-stimulated IP-10 test should compare with unstimulated IP-10 test, and more relative studies should be developed.

The types of included studies were cohort, case-control and cross-sectional studies. They were retrospective studies. Although the study design was not an important source of inconsistency (P = 0.9709), the specificity was higher with case-control when compared with cohort studies (88% vs 76%). In case-control studies, the presented results may be overestimated than the real results. We need more studies about these three types to explain the different results.

The overall performance with HIV-infected individuals was higher than HIV-noninfected and not reported individuals (81% vs 70% and 77%, 90% vs 87% and 68%), which is consistent with the previous studies^33–35^. In this meta-analysis, only 2 studies in HIV infected populations were included, both with small sample sizes. Besides, the confidence intervals of the diagnostic accuracy estimates for the HIV-infected subgroup are wide and overlap with the HIV negative studies. Although we agree with the result, there still need more related studies to support the results.

Certainly, this meta-analysis has several limitations. First, the sensitivity of IP-10 was 72% which didn’t meet the WHO TPP ‘minimum’ requirements (sensitivity >90%), it couldn’t be used as a rule out test for discriminating ATB from LTBI alone. When IP-10 test combines with other tests, the incremental benefit should be addressed. Furthermore, other issues such as poor reporting, laboratory infrastructure and expertise with IP-10 technology might lead analyse difficultly. Second, some studies included ATB and LTBI individuals after using chemotherapeutic agents, while others were not. This might have influenced the accuracy of IP-10 and increased the instability of participants. Third, the heterogeneity was a concern. Even though the World Bank income classification, study design, HIV-infected condition, cut-off and IP-10 condition were not significant sources of inconsistency (P > 0.05), they could also increase the inconsistency and reduce the stability of the whole outcomes. Besides, the intercurrent diseases (intercurrent disease, end-stage renal disease and liver cirrhosis) in the included studies might influence heterogeneity. Fourth, publication bias couldn’t be ignored. Because of the limited linguistic abilities, we included only English or Chinese studies. The real value of IP-10 for discriminating ATB from LTBI might lower than we report.

## Conclusion

This meta-analysis shows that IP-10 might be a potential marker for differentiating ATB from LTBI. The diagnostic accuracy of IP-10 is not influenced by its condition. Furthermore, multi-center, large and prospective studies are requested to support this finding.

## Method

### Literature search

We followed the Preferred Reporting Items for Systematic Reviews and Meta-Analyses criteria (PRISMA)^36^ English databases (PubMed, Web of Science, Embase, the Cochrane Library) and Chinese databases (CNKI, Wanfang, VIP, CBM) were used to search related citations up to January 2018. The language was restricted in English and Chinese. The search terms included “tuberculosis”, “active tuberculosis”, “latent tuberculosis” and “interferon gamma-induced protein 10”. A comprehensive literature search strategy which based on the following combination of MeSH terms and title/abstracts was utilised for PubMed database: (((((“Tuberculosis”[Mesh]) OR (((((((tuberculosis[Title/Abstract]) OR mycobacterium tuberculosis[Title/Abstract]) OR TB[Title/Abstract]) OR tuberculoses[Title/Abstract]) OR mycobacterium tuberculosis Infection*[Title/Abstract]) OR tuberculosis infection*[Title/Abstract]) OR active tuberculosis[Title/Abstract]))) OR ((((((Latent Tuberculoses[Title/Abstract]) OR Latent Tuberculosis[Title/Abstract]) OR latent tuberculosis infection*[Title/Abstract]) OR LTBI[Title/Abstract])) OR “Latent Tuberculosis”[Mesh]))) AND ((“Chemokine CXCL10”[Mesh]) OR ((((((Cytokine IP 10 Protein[Title/Abstract]) OR IP-10[Title/Abstract]) OR interferon gamma-induced protein 10[Title/Abstract]) OR interferon-inducible protein 10[Title/Abstract]) OR CXCL10[Title/Abstract]) OR Chemokine CXCL10[Title/Abstract])). Additionally, we manually looked for the reference lists of the applicable articles and reviews to find other potentially eligible studies.

### Inclusion and exclusion criteria

Studies reporting IP-10 for the discrimination of ATB from LTBI were included according to the following criteria: (1) evaluation the diagnostic performance of IP-10 for differentiating ATB from LTBI; (2) reporting on individuals with TB including ATB or LTBI (population); (3) provision of IP-10 in plasma or the whole blood as the index test and culture, clinical, radiological, TSTs and interferon-gamma tests as gold standard; (4) the primary outcomes including differential diagnostic performance of IP-10 (sensitivity and specificity); (5) randomized controlled trails, prospective and retrospective studies included (study design); (6) more than 5 patients reported meeting the inclusion criteria. We selected the most comprehensive research even though it was published two or three times. Studies not published in English and Chinese, other letters (except research letters), conference abstracts, veterinary experiments and case reports less than 5 individuals were excluded. Two investigators independently determined the obtained literature eligibility.

### Data extraction

The data were extracted including the first author, published time, country, world bank income classification, TB incidence rate per population (/100000), participants (ATB patients and LTBI subjects), the condition of index test (IP-10), diagnostic reference standard, study design, HIV-infected condition, cut-off value, sensitivity, specificity, true positive (TP: ATB patients with IP-10 value above the cut-off), false positive (FP: LTBI controls with IP-10 value above the cut-off), false negative (FN: ATB patients with IP-10 below the cut-off), and true negative (TN: LTBI controls with IP-10 value below the cut-off). Two investigators independently extracted data from selected articles, and disagreements were settled by discussing and reaching a consensus.

### Quality assessment

According to the Quality Assessment of Diagnostic Accuracy Studies tool-2 (QUADAS-2) recommended by the Cochrane Collaboration, two investigators independently reviewed the methodological quality of eligible articles^37^. The QUADAS-2 evaluated the risk of bias and applicability of eligible studies across four domains: patient selection, index test, reference standard and flow and timing. Selection bias exists in participants. In index test part, whether the participants detected in blind ways is critical. Information and disease progression bias are related to reference standard^36^. Signalling questions were included to help judge the quality of eligible articles^36^. Under the circumstance of disagreements, they were resolved by consensus.

### Statistical analysis

We used spearman correlation analysis to distinguish whether the threshold effect exist or not, and P > 0.05 indicated no threshold effect in this study. Then, Heterogeneity was calculated by evaluated by I^2^ and/or Cochrane Q test (I^2^ = 100% × (Q − df)/Q)^36^. I^2^ < 50%/P > 0.1 suggested using a fixed effect model; I^2^ > 50%/P < 0.1 indicated the inconsistency cannot be ignored and a bivariate random effects model should be utilized.

Meta-Disc (version 1.4) software was used to pool the primary diagnostic data^38^. The main outcomes evaluated were the discriminating ability of IP-10 for ATB from LTBI, The pooled sensitivity, specificity, positive likelihood ratio (PLR), negative likelihood ratio (NLR) and diagnostic odds ratio (DOR) were calculated^39^. DOR, a measure for overall accuracy of index test, could also be calculated by the formula “DOR = (TP/FN)/(FP/TN)”. We constructed the summary receiver operating characteristic (SROC) curve and calculated the area under the curve (AUC), which was a measure of differential diagnosis accuracy of index test^40,41^. An AUC less than 0.75 mean that IP-10 had a “not accurate” discriminate accuracy, between 0.75 and 0.93 mean that IP-10 had a “good” discriminate accuracy, and more than 0.93 mean that IP-10 had an “excellent” discriminate accuracy.

Additionally, we conducted meta-regression analysis to find possible sources of heterogeneity, and the subgroups including world bank income classification for countries (high-income vs. upper-middle-income), the condition of IP-10 (TB Ag-stimulated/unstimulated), the study design (cohort/case-control/cross-sectional), the HIV-infected condition (yes/no) and the cut-off of IP-10 (more than 2000/less than 2000 pg/ml). With respect to publication bias, Deeks’ funnel plots could be used to assess it^42^. The Stata (version 14.0) software was run with the “midas” command.
